# Sarcopenia and satellite cell homeostasis disruption: the dual function of NAD+ metabolism

**DOI:** 10.3389/fnut.2026.1803560

**Published:** 2026-06-05

**Authors:** Chenyuan Li, Yue Ai, Jieni Yan, Shuhua Wu

**Affiliations:** 1 Department of Geriatrics, The Second Affiliated Hospital of Soochow University, Suzhou, Jiangsu, China; 2 Department of General Medicine, The Second Affiliated Hospital of Soochow University, Suzhou, Jiangsu, China

**Keywords:** MuSC homeostasis, NAD+, sarcopenia, satellite cell, sirtuins

## Abstract

Sarcopenia is an age-related syndrome characterized by progressive loss of skeletal muscle mass and function, which is closely associated with impaired regenerative capacity of muscle satellite cells (MuSCs). During aging, the MuSC niche undergoes severe deterioration, including mitochondrial dysfunction, chronic inflammation, and neuromuscular junction (NMJ) degeneration, all of which compromise MuSC quiescence, proliferation, and differentiation.

Nicotinamide adenine dinucleotide (NAD+) serves as a critical coenzyme and signaling molecule that governs MuSC homeostasis in a context-dependent, dual-function manner. Moderate NAD+ repletion via precursors such as nicotinamide mononucleotide (NMN) or nicotinamide riboside (NR) activates SIRT1 and SIRT3, enhances mitochondrial bioenergetics, reduces oxidative stress, and promotes MuSC proliferation and myogenic differentiation. In contrast, under pathological or aging conditions, excessive or dysregulated NAD+ signaling activates SIRT2 to deacetylate PAX7 and repress Myogenic Differentiation 1 (MyoD), leading to cell-cycle arrest and MuSC exhaustion.

This review adopts a hypothesis-driven framework to systematically summarize the molecular crosstalk between NAD+ metabolism, sirtuin family deacetylases (SIRTs), and MuSC fate regulation. We integrate evidence from nearly 60 representative preclinical and clinical studies, clarify the dual-function role of NAD+, and address current inconsistencies in the field. We also highlight key limitations and propose future directions for developing NAD+-targeted therapies for sarcopenia.

## 1 Introduction

Sarcopenia is a progressive geriatric syndrome defined by the gradual loss of skeletal muscle mass, strength and physical performance. It is associated with a series of adverse outcomes including falls, fractures, disability, diminished quality of life, and increased mortality (1–3). Globally, sarcopenia affects 5% to 13% of adults aged 60 years and older, and the prevalence increases to 50% among individuals aged 80 years and above (2, 3). In China, the rapidly aging population has made sarcopenia a major public health challenge (4, 5).

Skeletal muscle satellite cells (MuSCs), the resident stem cells responsible for postnatal muscle growth and regeneration, are central to sarcopenia progression. Impairment of MuSC function and inhibition of myogenic differentiation are central drivers of sarcopenia. These defects arise primarily from aging-related deterioration of the MuSC

niche, including extracellular matrix stiffening, mitochondrial ROS accumulation, and elevated proinflammatory cytokine signaling (6–8). Such environmental changes suppress key myogenic pathways, including YAP/TAZ overactivation that represses myogenic differentiation, PI3K-Akt inhibition, and NF-κB-mediated repression of MyoD, ultimately blunting muscle repair and accelerating atrophy (9–11).

Emerging evidence identifies NAD+ decline as a central metabolic hallmark of aged MuSCs (12–14). NAD+ is indispensable for energy metabolism, DNA repair, and sirtuin-dependent deacetylation (15–17). Importantly, NAD+ exhibits a dual regulatory role in MuSC homeostasis: it supports regeneration under physiological conditions but reduces stemness maintenance and self-renewal under excessive or dysregulated conditions (18, 19).

**Figure 1:**
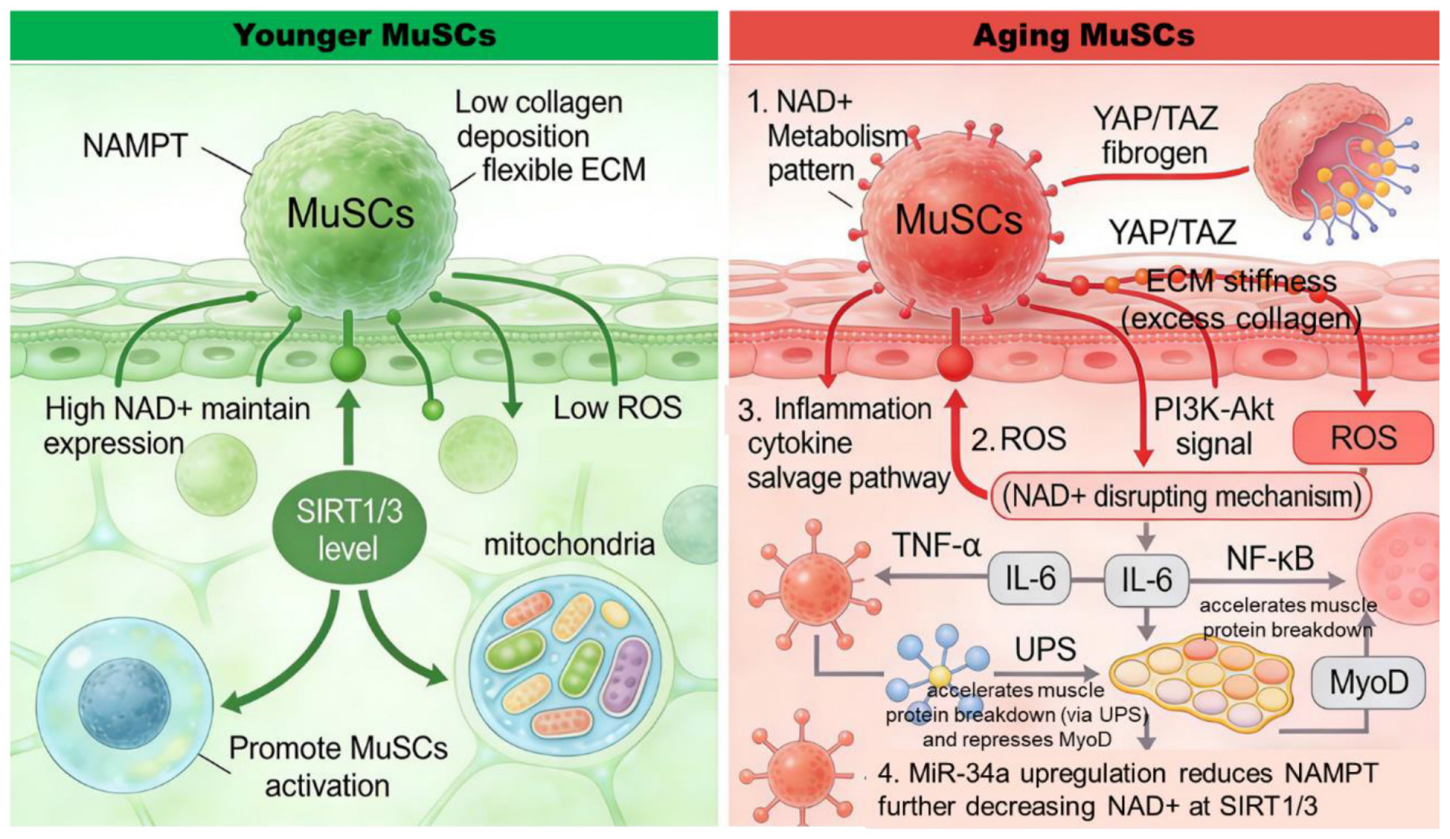
Schematic of MuSC Niche Deterioration in Aging and Its Impact on NAD+ Metabolism. Left Panel (Young): Quiescent MuSCs reside in a flexible ECM (low collagen deposition) with functional mitochondria (low ROS) and minimal proinflammatory cytokines. NAMPT expression is high, sustaining NAD+ levels via the salvage pathway. SIRT1/3 are activated, promoting MuSC quiescence and regenerative capacity. Right Panel (Aged): (1). ECM stiffness (excess collagen) activates YAP/TAZ, shifting MuSCs to fibrogenesis. (2). Mitochondrial dysfunction increases ROS, inhibiting PI3K-Akt signaling and reducing NAMPT (NAD+ salvage pathway impairment). (3). Proinflammatory cytokines (TNF-α, IL-6) activate NF-κB, which accelerates muscle protein breakdown (via UPS) and represses MyoD. (4). miR-34a upregulation further reduces NAMPT, depleting NAD+ and inactivating SIRT1/3.

This review is structured around a central hypothesis: NAD+ exerts dose-dependent, subcellular compartment-dependent, and sirtuin isoform-specific dual control over MuSC homeostasis. SIRT1 and SIRT3 primarily mediate pro-regenerative effects, whereas SIRT2 drives inhibitory outcomes (20, 21). Figure 1 visually illustrates the deterioration of the MuSC niche during aging and its direct impact on NAD+ homeostasis.

## 2 NAD+ metabolism: biosynthesis, degradation, and compartmentalization

NAD+ is a pivotal coenzyme involved in glycolysis, oxidative phosphorylation, and multiple signaling pathways (16, 22). Intracellular NAD+ levels decline progressively during aging, contributing to MuSC dysfunction (12–14).

### 2.1 NAD+ biosynthesis pathways

#### 2.1.1 De novo synthesis pathway

The *de novo* pathway synthesizes NAD+ from tryptophan or nicotinic acid (NA). This pathway is inefficient in skeletal muscle and MuSCs and plays a minor role (23, 24). The rate-limiting enzyme is quinolinic acid phosphoribosyltransferase (QPRT) (12, 24).

#### 2.1.2 Salvage Synthesis Pathway

The salvage pathway is the dominant source of NAD+ in MuSCs (12, 25, 26), including: (1) From nicotinamide (NAM): NAM → nicotinamide mononucleotide (NMN) (via nicotinamide phosphoribosyltransferase, NAMPT) → NAD+ (via nicotinamide mononucleotide adenylyltransferase, NMNAT) (25, 27, 28). (2) From nicotinamide riboside (NR): NR → NMN (via nicotinamide riboside kinase, NRK1/2) → NAD+ (26, 29, 30). NAMPT is the rate-limiting enzyme, and its expression is repressed by miR-34a during aging, leading to NAD+ depletion (31, 32). In contrast, NRK1/2 expression is relatively preserved during aging and can be further upregulated by SIRT1-activated PGC-1α, enabling effective utilization of exogenous NR to replenish NAD+.

**Figure 2:**
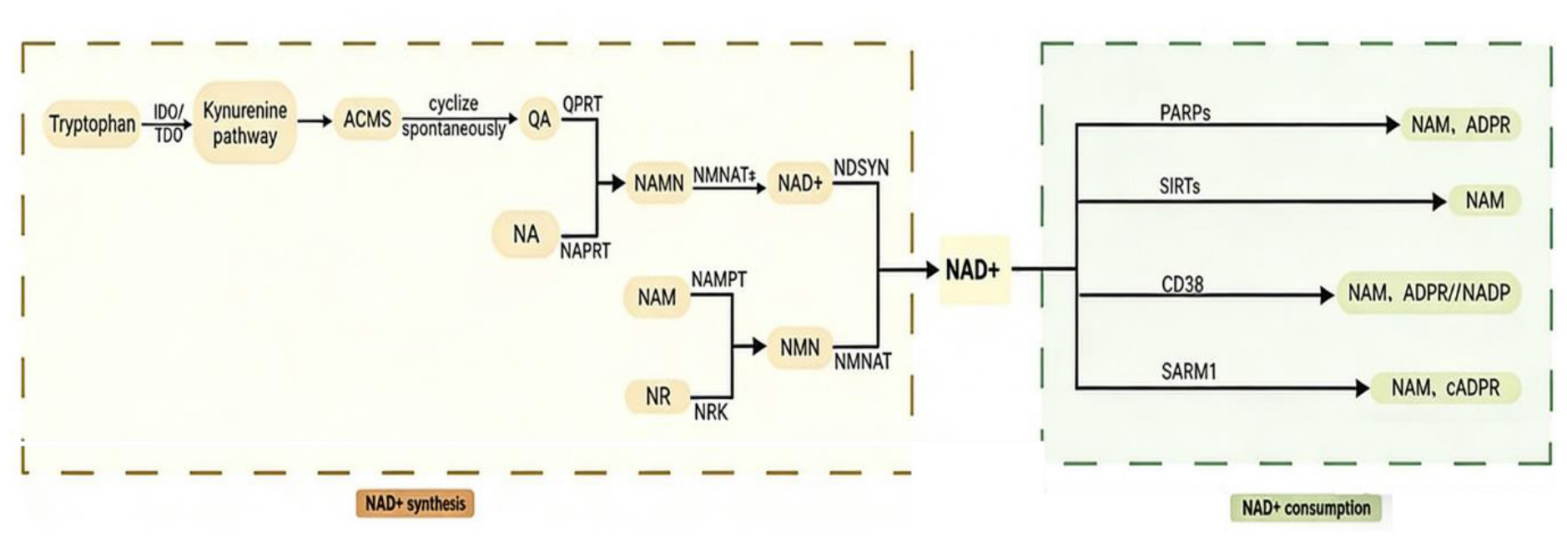
The synthesis of NAD+ primarily occurs through two pathways: the de novo synthesis pathway and the salvage pathway. Major NAD+ consumption enzymes (SIRTs, PARPs, CD38, SARM1) and their breakdown products are also illustrated.

Table 1 summarizes the core features of the two NAD+ biosynthesis pathways, highlighting why the salvage pathway is the dominant source of NAD+ in MuSCs and skeletal muscle. Figure 2 illustrates two pathways of synthesis of NAD+: the *de novo* synthesis pathway and the salvage pathway.

**Table 1:** Key features of NAD+ biosynthesis pathways in skeletal muscle and MuSCs.

Feature	De novo synthesis pathway	Salvage synthesis pathway
Primary tissues/Cells	Liver, immune cells; inefficient in MuSCs	Skeletal muscle, MuSCs (dominant pathway)
Subpathways	1. Tryptophan → Kynurenine → quinolinic acid (QA) → NAMN 2. NA → NAMN	1. NAM → NMN (via NAMPT) → NAD+2. NR → NMN (via NRK) → NAD+
Rate-Limiting enzyme	QRPT (quinolinic acid phosphoribosyltransferase)	NAMPT (nicotinamide phosphoribosyltransferase)
Energy requirement	High (requires tryptophan catabolism)	Low (recycles NAD+ degradation products)
MuSC-Specific regulation	Reduced indoleamine 2,3-dioxygenase (IDO) and tryptophan 2,3-dioxygenase (TDO) activities in aged MuSCs limits flux	- miR-34a (age-upregulated) targets NAMPT -PGC-1α (SIRT1-activated) upregulates NRK1
Precursor efficacy for MuSCs	NA (limited); tryptophan (negligible)	NMN, NR (high—directly boost NAD+ levels)

### 2.2 NAD+ degradation pathways

NAD+ is continuously degraded by three major enzyme families: (1) Sirtuins: consume NAD+ during deacetylation (16, 20); (2) Poly(ADP-ribose) polymerases (PARPs): mediate DNA repair and NAD+ hydrolysis (15, 17); (3) CD38/sterile alpha and TIR motif-containing 1 (SARM1): major NAD+ hydrolases; highly activated in aged tissues (28). CD38 is a primary driver of age-related NAD+ decline in muscle (28). Major NAD+ degradation products including nicotinamide (NAM) are continuously recycled back to NAD+ via the salvage pathway, where NAMPT catalyzes the rate-limiting step to form NMN, maintaining intracellular NAD+ homeostasis.

### 2.3 NAD+ compartmentalization

NAD+ pools in the nucleus, cytoplasm, and mitochondria are relatively independent (16, 33). Mitochondrial NAD+ regulates SIRT3 and respiration (34, 35). Nuclear NAD+ regulates SIRT1 and transcription (36, 37). Cytosolic NAD+ supports glycolysis, ATP production, and redox balance, linking cellular metabolic status to MuSC fate decisions. Aging disrupts compartmentation, contributing to the dual-function effects of NAD+ (13, 17).

### 2.4 NAD+ precursor dosing: preclinical and clinical considerations

Dosing regimens for NAD+ precursors vary widely across studies and different preclinical animal models (18, 38). (1) Mice: C57BL/6 aging mice, NAMPT-deficient, SIRT1–/–, SIRT3–/–, SIRT2–/– genetic models, muscle injury and sarcopenia models; common doses: NR 200–500 mg/kg/day, NMN 300–500 mg/kg/day (18, 38–40). Observations: Common effective doses were NR 200–500 mg/kg/day and NMN 300–500 mg/kg/day; moderate NAD+ repletion within this range activated SIRT1/3 and promoted MuSC regenerative capacity, while excessive dosing trended toward SIRT2 overactivation. Conclusions: Dose-dependent effects exist in mice, and moderate precursor supplementation is safe and pro-regenerative, whereas high doses may induce inhibitory effects on MuSC homeostasis. (2) Rats: Aging and denervation-induced muscle atrophy models (38). Observations: NAD+ precursors effectively reversed muscle atrophy and restored MuSC function in aged/denervated rats. Conclusions: NAD+ replenishment is effective in mitigating rodent muscle atrophy linked to aging or denervation, supporting the translatability of NAD+ targeted interventions across rodent species. (3) Non-human primates: Limited evidence supports age-related NAD+ decline in skeletal muscle (13). (4) In humans, typical doses range from 250–1,000 mg/day for NMN and 1,000 mg/day for NR (40, 41). High-dose NAD+ elevation may shift signaling toward SIRT2 activation, resulting in inhibitory effects on MuSCs (18, 42, 43). No consensus exists on optimal timing, duration, or formulation (38).

## 3 Dual regulation of MuSC homeostasis by NAD+

### 3.1 Pro-regenerative function of NAD+

Under physiological conditions, acute injury, or moderate supplementationwith NAD+ precursors (NMN, NR), NAD+ promotes MuSC regeneration (19, 39, 43). Four mechanisms have been reported: (1) SIRT1 deacetylates PGC-1α to enhance mitochondrial biogenesis; SIRT1-mediated deacetylation of PAX7 stabilizes PAX7 transcriptional activity, maintains MuSC stemness, and supports MuSC proliferation and early myogenic commitment (36, 44, 45); (2) SIRT1 activates FOXO3 to upregulate antioxidant genes (46, 47); (3) SIRT1 deacetylates p53 to reduce cellular senescence (48, 49); (4) SIRT3 enhances mitochondrial antioxidant capacity and reduces ROS (34, 50, 51). These events collectively improve MuSC proliferation, differentiation, and survival (19, 37).

**Implication for sarcopenia:** By sustaining MuSC regenerative potential, moderate NAD+ repletion helps preserve muscle mass and function, thereby delaying the onset and slowing the progression of sarcopenia (38, 52, 53).

### 3.2 Inhibitory function of NAD+

**Figure 3:**
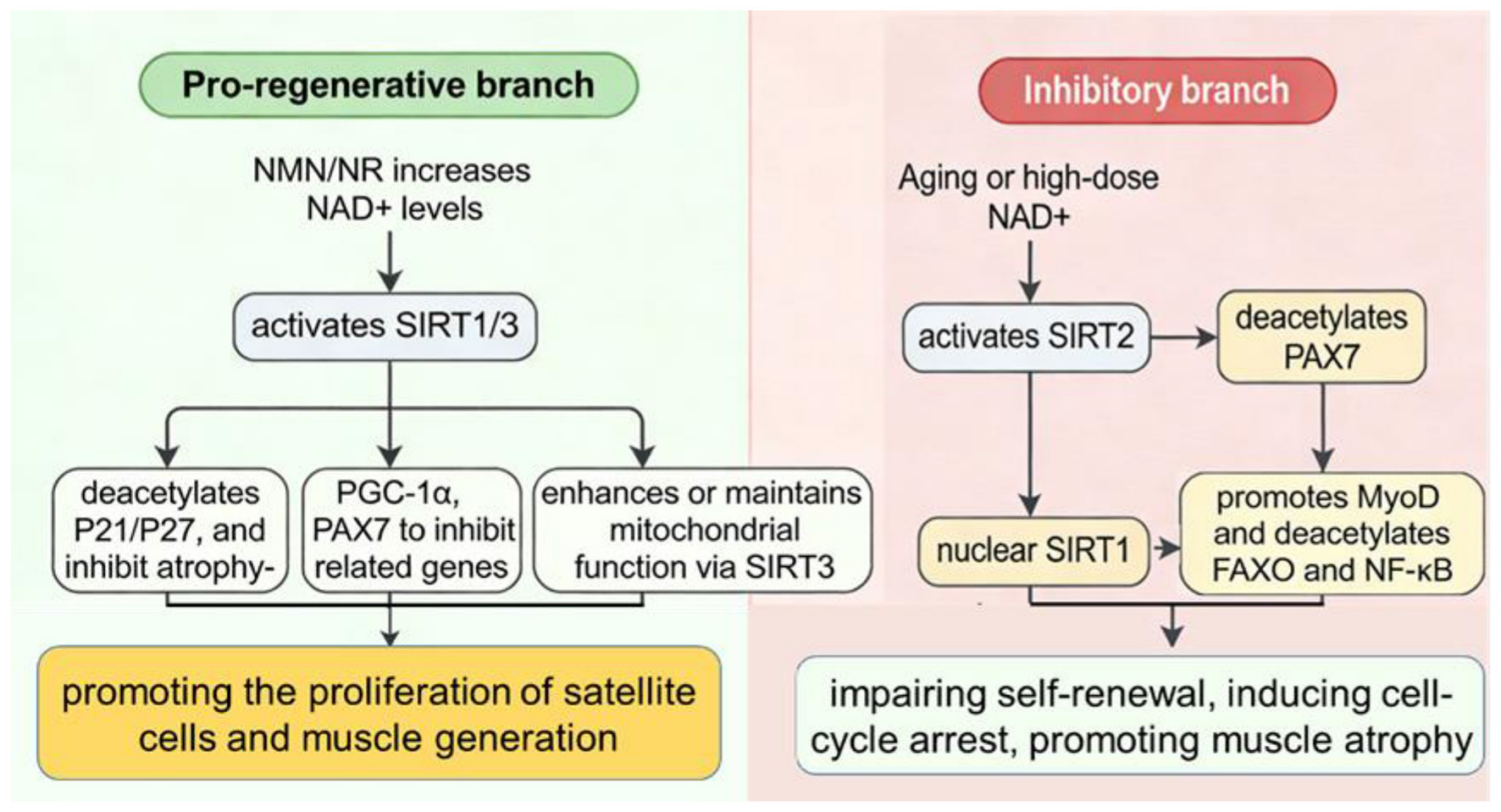
NAD+ metabolism affects muscle satellite cells (MuSCs) through the SIRT family, which is manifested in two pathways: (1) Proliferation-promoting pathway: NMN/NR increases NAD+ levels → activates SIRT1 → deacetylates PGC-1α, P21/P27, and PAX7 at distinct lysine residues to stabilize its transcriptional activity, sustain stemness, and promote MuSC proliferation. Meanwhile, SIRT3 enhances mitochondrial function, thereby facilitating muscle regeneration. (2) Inhibitory pathway: Excessive NAD+ activates SIRT2 to deacetylate PAX7 at other key lysine sites to abolish myogenic promoter binding and impair self-renewal; dysregulated SIRT1 represses MyoD, induces cell cycle arrest, and reduces myogenic differentiation, leading to MuSC depletion and muscle atrophy.

Under aging, pathological niche conditions, or excessive NAD+ elevation, NAD+ signaling becomes detrimental (Figure 3) (18, 21, 54). Three mechanisms have been observed: (1) SIRT2 deacetylates PAX7 and impairs self-renewal (42); (2) Pathological nuclear SIRT1 represses MyoD and inhibits myogenic differentiation (37); (3) Upregulation of p21/p27 induces G1 cell-cycle arrest (36, 44).

Elevated p21/p27 blocks cell-cycle progression and directly represses MyoD transcriptional activity, further suppressing myogenic differentiation. The final outcome is MuSC exhaustion and failed regeneration (42, 54).

Acetylated PAX7 preferentially binds to promoters of myogenic homeobox genes including MyoD, Myf5, and Myogenin to maintain stemness and direct myogenic commitment. SIRT2-mediated deacetylation abolishes this binding and impairs MuSC self-renewal (42, 55).

Dysregulated NAD+-SIRT2 signaling drives premature MuSC exhaustion and myogenic arrest, which directly accelerates age-related muscle loss and functional decline in sarcopenia (38, 56, 57).

Table 2 systematically summarizes the context, mediating molecules, and cellular outcomes of NAD+'s bidirectional effects, providing a framework for understanding the mechanisms detailed in Sections 3.1 and 3.2.

**Table 2:** Dual regulatory role of NAD+ in MuSC homeostasis.

Function	Context	Sirtuin isoform	Target molecule	Cellular outcome	Physiological effect
Pro-regenerative	Youth, injury, moderate NMN/NR	SIRT1 (nuclear/cytosolic)	PGC-1α, FOXO3, p53	Enhances mitochondria; reduces ROS; inhibits senescence	Promotes MuSC proliferation & differentiation
		SIRT3 (mitochondrial)	SOD2, GPX	Reduces mitochondrial oxidative stress	Preserves MuSC stemness & survival
Inhibitory	Aging, high-dose NMN, inflamed niche	SIRT2 (cytosolic/nuclear)	PAX7	Reduces PAX7 acetylation; impairs self-renewal	Blocks MuSC maintenance
		SIRT1 (nuclear, excessive)	MyoD	Represses MyoD activity; induces p21/p27	Inhibits differentiation; causes cycle arrest

## 4 Sirtuin isoform-specific mechanisms

### 4.1 SIRT1 and SIRT3: pro-regenerative mediators

SIRT1 is predominantly nuclear and regulates MuSC quiescence, self-renewal, and myogenic commitment (37). By deacetylating PGC-1α, SIRT1 boosts mitochondrial biogenesis and energy metabolism (36, 44, 45). SIRT1 also deacetylates FOXO3 and p53 to reduce oxidative stress and cellular senescence (47, 48), supporting long-term MuSC function (19, 37).

SIRT3 is exclusively mitochondrial and maintains mitochondrial fitness, reduces ROS production, and stabilizes electron transport chain activity (34, 35, 50). By preserving mitochondrial health, SIRT3 sustains MuSC survival, proliferation, and differentiation potential (19, 37). Together, SIRT1 and SIRT3 form the pro-regenerative arm of NAD+ signaling (18, 19, 37).

### 4.2 SIRT2: inhibitory mediator

SIRT2 acts as the detrimental effector of dysregulated NAD+ signaling in MuSC (21, 54). SIRT1-mediated deacetylation stabilizes PAX7 activity to maintain stemness and promote proliferation, whereas SIRT2 deacetylates PAX7 reducing its transcriptional activity and impairing MuSC self-renewal and stemness maintenance (42). SIRT2 also represses MyoD and upregulates p21/p27, triggering cell-cycle arrest and blocking myogenic differentiation. SIRT2 overactivation under aging or high NAD+ conditions leads to MuSC exhaustion and failed regeneration (21, 54).

## 5 Translational challenges and future directions

Despite growing preclinical evidence demonstrating that NAD+ metabolism regulates MuSC function and muscle maintenance, direct evidence linking NAD+ deficiency to human sarcopenia progression remains limited. several critical challenges must be addressed to enable safe and effective clinical translation.

First, considerable inconsistency exists in NAD? precursor dosing and administration regimens across preclinical and clinical studies. Optimal doses, duration, timing, and formulation of NMN, NR, or other NAD? boosters remain poorly defined (18, 38–43). High-dose supplementation may even trigger inhibitory effects on MuSC function through SIRT2 overactivation, highlighting context-dependent dose–response relationships (18, 21, 54).

Second, no isoform-specific sirtuin modulators are clinically available. SIRT1/3 drive beneficial effects while SIRT2 drives inhibition; non-specific modulators risk off-target effects (20, 21).

Third, long-term safety and efficacy data in humans remain scarce. Most preclinical studies in mice and clinical studies are short-term (4–12 weeks), small-scale, or focused on metabolic outcomes rather than muscle mass, strength, or satellite cell activity (38, 41, 43).

Fourth, personalized strategies are lacking. Responses to NAD+ augmentation vary significantly with age, sex, baseline NAD+ levels, physical activity, nutritional status, and chronic comorbidities (14, 38).

**NAD+-based sarcopenia therapies:** Developing NAD+-targeted interventions for sarcopenia requires: (1) Defining safe, effective dose windows for NAD+ precursors that favor SIRT1/3 over SIRT2 (18, 19, 38); (2) Developing isoform-selective SIRT1/3 agonists and SIRT2 inhibitors (20, 21); (3) Combining NAD+ augmentation with anti-inflammatory agents or exercise-mimetic signaling to alleviate chronic inflammation, reduce ECM stiffness, and restore a youthful MuSC niche microenvironment (53, 58); (4) Conducting large, long-term randomized controlled trials with muscle mass, strength, and physical performance as primary endpoints (38); (5) Establishing precision intervention protocols stratified by age, sex, metabolic health, and disease status (14, 38). By addressing these challenges, NAD+-based strategies may be translated into evidence-based therapies to combat sarcopenia and improve healthspan in the aging population (13, 17, 38).

## 6 Conclusion

NAD+ acts as a double-edged sword in MuSC homeostasis. Moderate NAD+ repletion supports muscle regeneration via SIRT1/3, while excessive or dysregulated NAD+ signaling inhibits MuSC function via SIRT2. This dual-function model provides a unifying framework for understanding NAD+ biology in sarcopenia and guides the development of safe and effective therapies.

Strategies targeting precise NAD+ repletion, isoform-specific sirtuin modulation, and niche restoration may offer safe and effective interventions to delay sarcopenia progression and improve muscle health in the elderly.
